# Editorial Note: Prolonged immune alteration following resolution of acute inflammation in humans

**DOI:** 10.1371/journal.pone.0321981

**Published:** 2025-03-31

**Authors:** The PLOS One Editors

After this article [1] was published, concerns were raised about Fig S1A. Specifically, the LC-MS/MS chromatograms in Fig S1A, with different labelling, appear similar to Figs in [2–15]

In response, the corresponding author stated that Fig S1A does not report experimental results for the study reported in [1] and can be disregarded. Fig S1A was used for illustrative purposes only, to show the type of LC-MS/MS data of analytical standards produced by the Shimadzu LC-20AD HPLC and Shimadzu SIL-20AC autoinjector and QTrap 5500 (ABSciex, Warrington, UK) liquid chromatography-mass spectrometer instrumentation, as described in [16].

During editorial follow-up, another issue emerged regarding results reported in Fig 4.

The corresponding author reanalyzed the LC-MS/MS lipid data in [1] using MultiQuant software (ABSciex, Warrington, UK) with the result that the SPM values reported in Fig 4 were below the limits of quantification and Figs 4E-H should be disregarded. They stated that this concern did not apply to the prostanoid results. This reanalysis was reported in [17].

A preprint [18] by Jesmond Dalli, a co-author of [1], reported another reanalysis using methodologies to determine signal to noise ratios as reported in [19], and called into question the reliability of [17]. The last two authors of [1] expressed different views on comparing sensitivity and limits of quantitation of lipid mediators between [1] and [19] and D. Gilroy noted that the equipment used in [19] is different to that used in [1]. J. Dalli stands by the findings reported in [1].

SPM data presented in Fig 4 should be interpreted in the context of both [17] and [18] and the apparent disagreement within the author group regarding the reliability of these results. A member of the PLOS One Editorial Board stated that SPM results in Fig 4 are not essential in supporting the article’s main conclusions [1].

The Data Availability statement of [1] is updated to: The individual-level data underlying the remainder of the results can be found at https://zenodo.org/records/10532531.

Owing to the concerns about similarity with previously published content, including [2], published in 2016 by the National Academy of Sciences of the United States of America [2], which is not offered under a CC BY license, this article [1] was republished on March 17, 2025, to remove Fig S1. Interested readers can view the figure of concern in Fig 3A of [2].
